# Artificial intelligence-based volumetric analysis of muscle atrophy and fatty degeneration in patients with hip osteoarthritis and its correlation with health-related quality of life

**DOI:** 10.1007/s11548-022-02797-8

**Published:** 2022-12-26

**Authors:** Makoto Iwasa, Masaki Takao, Mazen Soufi, Keisuke Uemura, Yoshito Otake, Hidetoshi Hamada, Yoshinobu Sato, Nobuhiko Sugano, Seiji Okada

**Affiliations:** 1 Department of Orthopaedic Medical Engineering, Osaka University Graduate School of Medicine, Suita, Japan; 2 Department of Orthopaedic Surgery, Osaka University Graduate School of Medicine, 2-2 Yamadaoka, Suita, Osaka 565-0871 Japan; 3 Graduate School of Information Science, Nara Institute of Science and Technology, Ikoma, Japan

**Keywords:** Muscle fatty degeneration, Muscle atrophy, Osteoarthritis, Patient-reported outcome measures, Health-related quality of life

## Abstract

**Purpose:**

Artificial intelligence (AI) technologies have enabled precise three-dimensional analysis of individual muscles on computed tomography (CT) or magnetic resonance images via automatic segmentation. This study aimed to perform three-dimensional assessments of pelvic and thigh muscle atrophy and fatty degeneration in patients with unilateral hip osteoarthritis using CT and to evaluate the correlation with health-related quality of life (HRQoL).

**Methods:**

The study included one man and 43 women. Six muscle groups were segmented, and the muscle atrophy ratio was calculated volumetrically. The degree of fatty degeneration was defined as the difference between the mean CT values (Hounsfield units [HU]) of the healthy and affected sides. HRQoL was evaluated using the Western Ontario and McMaster Universities Osteoarthritis (WOMAC) index and the Japanese Orthopaedic Association Hip Disease Evaluation Questionnaire (JHEQ).

**Results:**

The mean muscle atrophy rate was 16.3%, and the mean degree of muscle fatty degeneration was 7.9 HU. Multivariate correlation analysis revealed that the WOMAC stiffness subscale was significantly related to fatty degeneration of the hamstrings, the WOMAC physical function subscale was significantly related to fatty degeneration of the iliopsoas muscle, and the JHEQ movement subscale was significantly related to fatty degeneration of the hip adductors.

**Conclusion:**

We found that fatty degeneration of the hamstrings, iliopsoas, and hip adductor muscles was significantly related to HRQoL in patients with hip osteoarthritis. These findings suggest that these muscles should be targeted during conservative rehabilitation for HOA and perioperative rehabilitation for THA.

## Introduction

Traditional assessments of muscle atrophy and fatty degeneration include two-dimensional (2D) assessments using cross-sectional images; however, these assessments vary depending on the location of the cross section or the position of the photographed limb [1]. Three-dimensional (3D) evaluation is suitable for eliminating differences in the cross-sectional position [2, 3]. However, in many cases, manual segmentation of the volumetric geometry of the muscles requires considerable effort, which makes it difficult to analyze multiple cases. Current artificial intelligence (AI) technologies have enabled us to perform precise 3D analysis of individual muscles on computed tomography (CT) or magnetic resonance imaging (MRI) by automatic segmentation. We have previously developed an AI-based system that segments the muscles of the pelvis and thigh on pelvic CT images, with an average surface difference accuracy of 0.994 mm and a Dice coefficient of 0.891 [4].

Patients with hip osteoarthritis (HOA) develop muscle atrophy and muscle fatty degeneration of the pelvis and thigh, accompanied by pain and limited range of motion [2, 5–10]. Recent studies using CT and MRI data reported that the degree of atrophy and fatty degeneration in patients with HOA varies according to muscle [2, 5–10]. Many studies have assessed muscle atrophy [2, 5, 7, 9, 11]; however, only a few studies have quantitatively assessed muscle degeneration [6, 8]. The volume of the muscle represents the demand for muscle power output and the muscle tends to show hypertrophy with increasing demand, and atrophy with decreasing demand [12]. However, fatty degeneration of the muscle complicates the interpretation of muscle volume, as it increases muscle volume but decreases contractility [13]. To assess the muscle, fatty degeneration needs to be considered in addition to the 3D volume.

A qualitative decrease in muscle mass is considered to decrease the health-related quality of life (HRQoL). It is unclear which muscles affected by atrophy or fatty degeneration would impair the HRQoL. Clarifying the influence of atrophy and fatty degeneration on the muscle with regard to HRQoL is useful for developing treatment strategies for patients with HOA.

This study aimed to perform a 3D assessment of pelvic and thigh muscle atrophy and fatty degeneration in patients with unilateral HOA using AI-based CT analysis and to evaluate the correlation of muscle atrophy and fatty degeneration with HRQoL.

## Methods

### Participants

Patients with unilateral HOA who underwent THA at our hospital were included in the study. We excluded patients with bilateral hip disease; a history of trauma, infection, or tumor in the pelvis or femur; a history of hip surgery; osteoarthritis of the knee; or a lack of preoperative CT scan. A total of 44 patients who consented to participate in the study and answered the questionnaire were included in the analysis. Unilaterality was defined as an asymptomatic, healthy side, with a joint space width of at least 2 mm [14]. The study patients included one man and 43 women. Demographic data are presented as mean ± standard deviation (range) for age, 65.5 ± 9.1 (47–85) years; height, 154.0 ± 6.6 (142.3–170.0) cm; body weight, 1, 58.5 ± 9.5 (41.0–82.0) kg; and body mass index, 24.6 ± 3.4 (19.1–31.2) kg/m^2^. The Kellgren and Lawrence (KL) grading system [15] was used to grade the HOA stage, with grade 3 noted in nine patients and grade 4 in 35 patients.

### Computed tomography

CT images were obtained preoperatively using a 64-slice CT scanner (Optima CT660 Pro; GE Healthcare, Milwaukee, WI, USA) according to the following protocol: voltage, 120 kV; current, 250 mA; helical pitch, 1.375:1; slice thickness, 1.25 mm; X-ray tube rotation speed, and 0.6 s. The patients were placed in the supine position, and limb imaging was performed in a relaxed position. The area from the iliac crest to the femoral condyle was included in the imaging.

### Image analysis

**Fig. 1:**
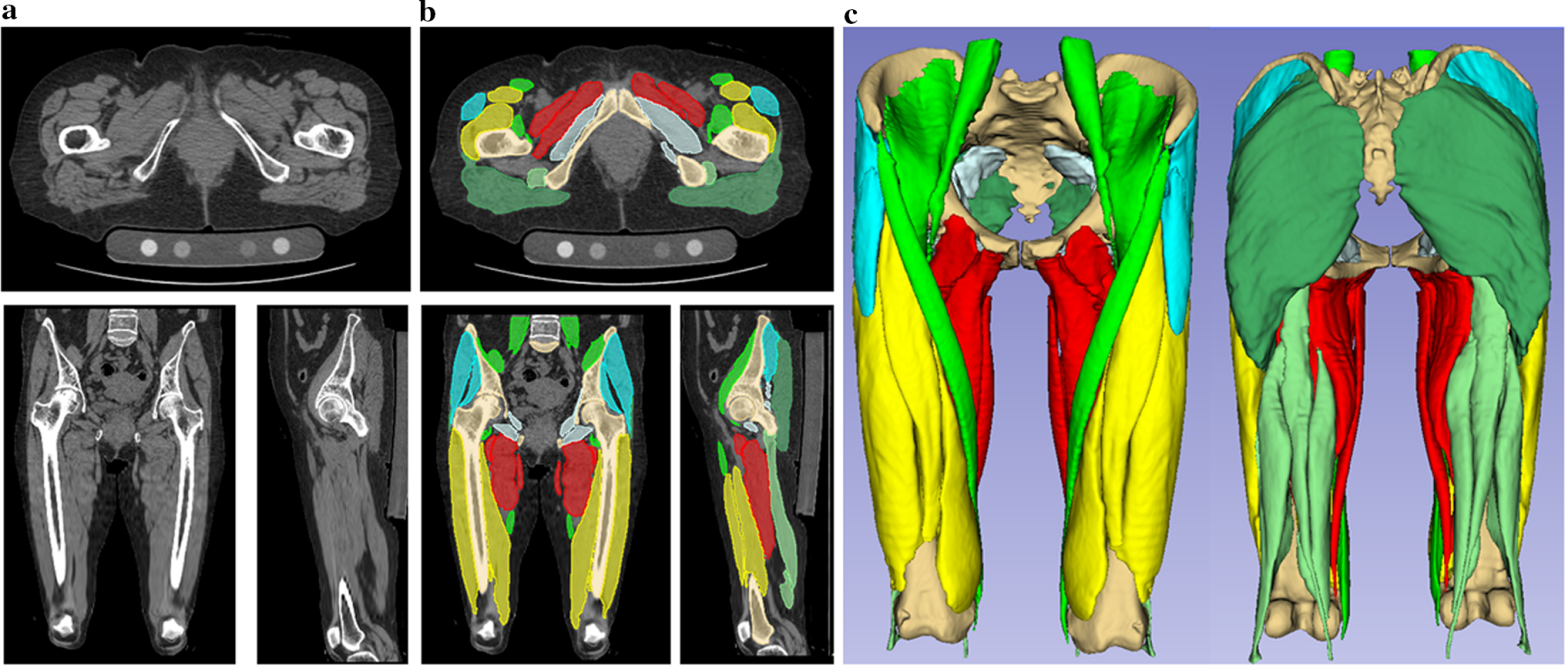
Computed tomography images of a patient with right hip osteoarthritis. **a** Original computed tomography images of muscle groups and bone regions; **b** segmentation images of muscle groups and bone regions; **c** three-dimensional models reconstructed from computed tomography images showing muscle atrophy and fatty degeneration on the affected side

The pelvic and thigh muscles were segmented using an AI-based method developed using a Bayesian U-Net model (average symmetric surface distance of 0.994 mm) [4]. An orthopedic surgeon (MI) specializing in musculoskeletal imaging reviewed the segmented muscles and confirmed their accuracy (Fig. 1). The pelvic and thigh muscles were arranged into the following groups according to their function: the gluteus maximus, gluteus medius and minimus, iliopsoas (iliacus and psoas), hip adductors (pectineus, adductor major, adductor longus, adductor brevis, and gracilis), quadriceps (vastus lateralis, vastus medialis, vastus intermedius, and rectus femoris), and hamstrings (semitendinosus, semimembranosus, and biceps femoris).

The muscle atrophy ratio was calculated by dividing the difference in the muscle volumes of the healthy and affected sides by the muscle volume of the healthy side and is expressed as percentage. The degree of muscle fatty degeneration was defined as the difference between the mean CT values of the muscle in the healthy and affected sides. Reportedly, the attenuation of a skeletal muscle, as determined by CT values (Hounsfield unit [HU]), is correlated with the lipid content of that muscle [16]. In previous reports, density values of 30 HU or more in CT slices were classified as pure muscle [17, 18]. However, no consensus on the cutoff value between muscle and fat is yet reached [18].

### HRQoL

HRQoL was evaluated using two types of patient-reported outcome measures: the Western Ontario and McMaster Universities Osteoarthritis (WOMAC) index [19] and the Japanese Orthopaedic Association Hip Disease Evaluation Questionnaire (JHEQ) [20]. The WOMAC score is a condition-specific measure of HRQoL for HOA and it comprises 24 items under three subscales. The three subscales are pain (five items), stiffness (two items), and physical function (17 items). A high score indicates severe pain, stiffness, and functional limitations (four is the worst score and zero is the best score). In contrast, the JHEQ is an evaluation method that includes the assessment of deep hip flexion motions. The JHEQ consists of 21 items under three subscales. The three subscales are pain (seven items), movement (seven items), and mental (seven items). A low score indicates severe pain, severe movement limitations, and poor mental health (zero is the worst score and four is the best score).

### Statistical analysis

After normality was assessed using the Shapiro–Wilk test, differences between groups were assessed using the paired Student’s *t* test and Wilcoxon signed-rank test. Correlations were assessed using Spearman rank correlation coefficient tests. Multiple regression analysis was performed to examine which muscle groups were associated with each subscale and the total score of HRQoL among the muscle groups that showed a statistically significant univariate correlation with HRQoL scores. Statistical analyses were performed using the JMP 15 software (SAS Institute Inc., Cary, NC, USA). Statistical significance was set at *p* < 0.05.

## Results

### Characteristics of muscle atrophy and fatty degeneration

A comparison of the pelvic and thigh muscle groups between the affected and healthy sides of patients with unilateral HOA revealed significant atrophy and fatty degeneration in all the groups (Table 1). The mean muscle atrophy ratio was 16.3% (range 13.2 –19.4%), and the mean degree of muscle fatty degeneration was 7.9 HU (range 5.6–11.5 HU).

**Table 1:** Atrophy ratio and fatty degeneration of the pelvic and thigh muscles of the affected side compared to the healthy side

	Muscle atrophy ratio (%)*^1^	*p* value*^2^	Degree of muscle fatty degeneration (HU)*^1^	*p* value*^3^
Gluteus maximus	17.9 ± 11.4	< 0.001	11.3 ± 7.2	< 0.001
Gluteus medius and minimus	15.3 ± 9.4	< 0.001	11.5 ± 7.5	< 0.001
Iliopsoas	19.4 ± 12.7	< 0.001	6.2 ± 5.4	< 0.001
Hip adductors	18.7 ± 10.1	< 0.001	6.8 ± 4.4	< 0.001
Quadriceps	13.2 ± 10.8	< 0.001	3.3 ± 3.2	< 0.001
Hamstrings	12.9 ± 8.6	< 0.001	5.6 ± 3.9	< 0.001

*^1^Values are expressed as mean ± standard deviation

*^2^The difference between the affected and healthy sides was assessed using paired Student’s *t* test

*^3^The difference between the affected and healthy sides was assessed using the Wilcoxon signed-rank test

### Univariate analysis of correlations of muscle atrophy and fatty degeneration with HRQoL

The mean total WOMAC score was 35.0 (range 5–67); the mean pain subscale score was 8.1 (range 2–15); the mean stiffness subscale score was 4.0 (range 1–6); and the mean physical function subscale score was 29.0 (range 7–50). The pain subscale score was not significantly correlated with the muscle atrophy ratio or the degree of fatty degeneration in any of the muscle groups. The stiffness subscale score was significantly correlated with the muscle atrophy ratio of the gluteus maximus and quadriceps and with the degree of muscle fatty degeneration of the gluteus maximus, iliopsoas, hip adductors, and hamstrings. The physical function subscale score was significantly related to the muscle atrophy ratio of the iliopsoas, hip adductors, quadriceps, and hamstrings and with the degree of muscle fatty degeneration of the gluteus maximus, iliopsoas, hip adductors, and hamstrings. The total WOMAC score was significantly related to the muscle atrophy ratio of the iliopsoas, hip adductors, quadriceps, and hamstrings and with the degree of muscle fatty degeneration of the gluteus maximus, iliopsoas, hip adductors, quadriceps, and hamstrings (Table 2).

**Table 2:** Correlation between the Western Ontario and McMaster Universities index and muscle atrophy/fatty degeneration

	Muscle group	Pain	*p* value*	Stiffness	*p* value*	Function	*p* value*	Total	*p* value*
Atrophy	Gluteus maximus	0.16	0.299	0.31	0.041	0.23	0.130	0.25	0.104
	Gluteus medius and minimus	0.01	0.937	0.08	0.628	0.20	0.196	0.17	0.261
	Iliopsoas	0.26	0.090	0.25	0.109	0.32	0.032	0.34	0.025
	Hip adductors	0.21	0.174	0.27	0.079	0.31	0.043	0.32	0.037
	Quadriceps	0.25	0.096	0.35	0.022	0.31	0.039	0.34	0.026
	Hamstrings	0.16	0.311	0.23	0.133	0.30	0.046	0.30	0.048
Fatty degeneration	Gluteus maximus	0.17	0.257	0.33	0.030	0.36	0.019	0.36	0.019
	Gluteus medius and minimus	0.01	0.941	0.08	0.589	0.26	0.087	0.23	0.141
	Iliopsoas	0.29	0.061	0.34	0.021	0.41	0.003	0.41	0.003
	Hip adductors	0.28	0.188	0.34	0.025	0.43	0.006	0.44	0.006
	Quadriceps	0.18	0.252	0.26	0.094	0.29	0.054	0.30	0.050
	Hamstrings	0.26	0.087	0.40	0.007	0.39	0.009	0.41	0.006

*The correlation coefficient and *p* value were calculated using the Spearman rank correlation coefficient test

The mean total JHEQ score was 26.0 (range 1–50); the mean pain subscale score was 9.0 (range 1–18); the mean movement subscale score was 6.3 (range 0–18); and the mean mental subscale score was 10.8 (range 0–28). The pain and mental subscale scores were not significantly related to any of the groups. The movement subscale score was significantly related to the atrophy ratio of the iliopsoas, hip adductors, and hamstrings and the degree of fatty degeneration of the gluteus maximus, iliopsoas, hip adductors, and hamstrings. The total JHEQ score was not significantly related to any muscle group (Table 3).

**Table 3:** Correlation between the Japanese Orthopaedic Association Hip Disease Evaluation Questionnaire (JHEQ) and muscle atrophy/fatty degeneration

	Muscle group	Pain	*p* value*	Movement	*p* value*	Mental	*p* value*	Total	*p* value*
Atrophy	Gluteus maximus	− 0.07	0.644	− 0.18	0.233	− 0.09	0.546	− 0.13	0.395
	Gluteus medius and minimus	− 0.12	0.448	− 0.17	0.276	0.01	0.978	0.01	0.539
	Iliopsoas	− 0.12	0.448	− 0.40	0.008	− 0.18	0.237	− 0.26	0.087
	Hip adductors	− 0.12	0.442	− 0.33	0.027	− 0.07	0.652	− 0.19	0.225
	Quadriceps	− 0.20	0.204	− 0.20	0.186	− 0.17	0.263	− 0.22	0.153
	Hamstrings	− 0.11	0.465	− 0.31	0.044	− 0.10	0.517	− 0.19	0.216
Fatty degeneration	Gluteus maximus	− 0.19	0.209	− 0.35	0.021	− 0.14	0.380	− 0.25	0.103
	Gluteus medius and minimus	− 0.06	0.683	− 0.23	0.129	0.09	0.573	− 0.06	0.708
	Iliopsoas	− 0.19	0.213	− 0.43	0.003	− 0.14	0.365	− 0.28	0.067
	Hip adductors	− 0.21	0.179	− 0.45	0.002	− 0.14	0.365	− 0.29	0.056
	Quadriceps	− 0.16	0.303	− 0.29	0.053	− 0.06	0.695	− 0.18	0.234
	Hamstrings	− 0.19	0.217	− 0.42	0.005	− 0.18	0.253	− 0.29	0.055

*The correlation coefficient and *p* value were calculated using the Spearman rank correlation coefficient test

### Multivariate analysis of correlations of muscle atrophy and fatty degeneration with HRQoL

The WOMAC stiff subscale was significantly related to the degree of fatty degeneration of the hamstrings, and the physical function subscale and total WOMAC score were significantly related to the degree of fatty degeneration of the iliopsoas. The JHEQ movement subscale score was significantly related to the degree of fatty degeneration of the hip adductors (Table 4).

**Table 4:** Multilinear regression analysis for evaluating the association between muscle atrophy/fatty degeneration and health-related quality of life

		Muscle group	*β*	95%CI	*p* value
WOMAC	Stiff	Hamstrings fatty degeneration	0.40	0.04–0.26	0.007
	Function	Iliopsoas fatty degeneration	0.43	0.42–1.98	0.003
	Total	Iliopsoas fatty degeneration	0.44	0.54–2.41	0.003
JHEQ	Movement	Hip adductors fatty degeneration	− 0.45	− 0.75 to − 0.18	0.002

*WOMAC* Western Ontario and McMaster Universities index, *JHEQ* Japanese Orthopaedic Association Hip Disease Evaluation Questionnaire, *β* standard regression coefficient, *CI* confidence interval

## Discussion

In this study, AI-based 3D assessment of the pelvic and thigh muscles of patients with unilateral HOA was performed to address the correlation of muscle atrophy and fatty degeneration with HRQoL. There was significant atrophy and fatty degeneration in all muscle groups on the affected side compared with that noted on the healthy side. Multivariate analysis showed that the degree of fatty muscle degeneration in the hamstrings, iliopsoas, and hip adductors was strongly related to HRQoL.

### Muscle atrophy and fatty degeneration in patients with HOA

The muscle atrophy ratio and degree of fatty degeneration in patients with HOA vary among studies. The discrepancy in the study findings is considered to be due to differences in the HOA stage across studies [2, 6–9]. Loureiro et al. volumetrically evaluated muscle atrophy using MRI in 12 unilateral HOA cases and seven bilateral HOA cases in patients with KL grade ≤ 3. Atrophy of the gluteus medius and gluteus minimus has not been reported [2]. Grimaldi et al. volumetrically evaluated muscle atrophy in 12 unilateral HOA cases (six mild [KL grades 1 and 2] and six advanced [KL grades 3 and 4] cases) and reported atrophy of the gluteus maximus, gluteus medius, and piriformis in advanced HOA cases but not in mild HOA cases [7, 9]. It was reported that no significant atrophy was observed in some muscles in HOA cases with low KL grade [2, 7, 9]. In our study, all the pelvic and thigh muscle groups showed significant atrophy and fatty degeneration, as 80% of the patients had KL grade 4. Thus, the cohort in our study might be appropriate for evaluating the correlation between muscle quality and HRQoL. In a study similar to ours, Rasch et al. evaluated muscle atrophy and fatty degeneration using CT in 22 cases of unilateral HOA before THA. Significant atrophy has been reported in the gluteus maximus, adductors, psoas major, rectus femoris, vastus femoris, and hamstrings. Similarly, significant fatty degeneration has been reported in the gluteus maximus, gluteus medius, gluteus minimus, adductors, vastus femoris, and hamstrings [8]. However, significant atrophy has not been reported in the gluteus medius and gluteus minimus [8]. The difference in muscle atrophy observed in our study may be due to the 2D method.

### Correlation between muscle atrophy and fatty degeneration and the WOMAC index

Our study found that the WOMAC stiffness subscale was significantly related to the degree of muscle fatty degeneration of the hamstrings. The hamstrings are biarticular muscles that function a hip extensors and knee flexors. This suggests that the hamstrings simultaneously function as agonists and antagonists to allow simultaneous hip and knee extensions [21]. Decreased hamstring flexibility impairs hip motion [22] and, therefore, may be related to hip stiffness. In addition, fatty degeneration of muscles can cause stiffness owing to decreased flexibility [23]. However, it had been suggested that muscle atrophy was not related to flexibility [23]. Hamstrings with fatty degeneration are less flexible, and the affected patients may be conscious of hip stiffness during some daily activities.

The physical function subscale was related to the degree of fatty degeneration of the iliopsoas. The iliopsoas muscle connects the trunk to the femur [24]. Due to the anatomical characteristics, it acts as a hip flexor and stabilizes the hip joint and trunk [24]. Therefore, it plays an important role in most daily activities, such as posture control, walking, and running, and is of increasing interest as a health biomarker [25, 26]. One study suggested that fatty degeneration of the iliopsoas is related to external rotation of the femur and may reduce hip stability in patients after revision THA [27]. Fatty degeneration of the iliopsoas in patients with end-stage HOA may impair hip stability and flexor strength output and affect their activities of daily living.

### Correlation between muscle atrophy and fatty degeneration and the JHEQ

The JHEQ movement subscale was found to be significantly related to the degree of fatty degeneration of the hip adductors through multivariate analysis. The hip adductors occupy a relatively large share of the thigh muscles [28, 29], and as an assembly of regions with different functions [30], they contribute to hip motion and hip stabilization during movement [31]. The hip adductors act as hip flexors in the shallow hip flexion position and as hip extensors in the deep hip flexion position, in addition to the adduction motion of the hip joint [32, 33]. It has been suggested that hip extension torque during deep hip flexion is greater than that in other hip extensors such as the gluteus maximus and hamstrings [33, 34]. Thus, the hip adductors are reported to be greatly activated during squatting, which is a typical deep hip flexion movement [35, 36]. The hip adductor muscles are particularly important for patients with reduced knee extensor strength and for the elderly because they are dependent on hip extension torque to compensate for an increased lateral sway [37, 38] and on hip intrinsic sensation to maintain standing balance [39]. The JHEQ movement subscale reflects the lifestyle of Asian people who require deep hip flexion [20]. Therefore, it is possible that the hip adductor muscles, which are important for deep hip flexion motion, are significantly related to the JHEQ movement subscale score in patients with end-stage HOA.

Peirisr et al. investigated the relationship between HRQoL and muscle changes in patients with HOA and reported that muscle atrophy of the hip adductors was significantly related to hip disability and osteoarthritis outcome score [40]. However, they did not evaluate the relationship with muscle fatty degeneration [40]. The univariate correlation analysis of the current study revealed that the muscle atrophy ratio and degree of muscle fatty degeneration of the hip adductors were both correlated well with the physical function subscales of the WOMAC, and movement subscales of the JHEQ. We evaluated muscle fatty degeneration, and multivariate analysis showed that muscle fatty degeneration of the hip adductors was more strongly related to HRQoL than to muscle atrophy.

Muscle volume is reduced with atrophy but can increase with fatty degeneration [13]. This indicates that the muscle atrophy ratio can be masked by fatty degeneration. Therefore, caution must be exercised when interpreting muscle volume results. A recent study of patients with knee OA found that knee extensor strength was not related to quadriceps muscle volume; however, it was related to intramuscular fat volume, and more intramuscular fat was related to decreased knee extensor strength [41]. To evaluate muscles in patients with HOA, it is recommended to evaluate not only the volume but also the degree of fatty degeneration, which is strongly related to HRQoL.

Exercises such as eccentric and concentric training have been reported to improve fatty muscle degeneration [42–46]. Targeted training of the hamstrings, iliopsoas, and hip adductor muscles identified in this study may improve fatty degeneration and be useful to improve HRQoL.

### Limitations

This study has several limitations. First, only 44 patients were included in this study. Therefore, we were unable to investigate how the duration from the onset of hip symptoms and comorbidities influences the muscle atrophy ratio and degree of fatty degeneration. This study is a preliminary report to examine how muscle atrophy and fatty degeneration, evaluated using the 3D method, correlate with HRQoL. Second, there was no control group in this study because of the difficulty in obtaining CT images of healthy subjects. We calculated the atrophy ratio and degree of fatty degeneration by comparing the affected side to the healthy side in unilateral HOA. The advantage of this method is that factors such as physical characteristics, age, and sex can be generalized. Finally, the combined effect of muscle atrophy and fatty degeneration on HRQOL was not evaluated due to the small sample size of this pilot study. Further study of larger sample size would be necessary.

## Conclusion

Muscle atrophy and fatty degeneration of all pelvic and thigh muscles were significant in patients with HOA before THA. Fatty degeneration of the hamstrings, iliopsoas, and hip adductors was significantly associated with HRQoL. These findings suggest that these muscles should be targeted during conservative rehabilitation for HOA and perioperative rehabilitation for THA.

## Data Availability

Data sharing is not applicable to this article as no datasets were generated or analyzed during the current study.
